# Spatial and Temporal Variability of Trace and Macro Elements in the Red Crab *Pleuroncodes planipes* in the Pacific Coast of the Baja California Peninsula, Mexico

**DOI:** 10.3390/ani13050822

**Published:** 2023-02-24

**Authors:** Juan Antonio De Anda-Montañez, Tania Zenteno-Savín, Eduardo F. Balart, Baudilio Acosta-Vargas, Ramón Gaxiola-Robles, Lia Celina Méndez-Rodríguez

**Affiliations:** 1 Centro de Investigaciones Biológicas del Noroeste, S.C. (CIBNOR), Calle Instituto Politécnico Nacional No. 195, Col. Playa Palo de Santa Rita Sur, La Paz 23096, Baja California Sur, Mexico; 2 Hospital General de Zona No.1, Instituto Mexicano del Seguro Social, 5 de Febrero y Héroes de la Independencia, Col. Centro, La Paz 23000, Baja California Sur, Mexico

**Keywords:** crustacean, marine economic resource, health risk, marine pollution, El Niño Southern Oscillation

## Abstract

**Simple Summary:**

The content of trace and macro elements may vary between fishery products. Although several trace and macro elements are essential for life, they can adversely affect health at concentrations above the requirements of a species. The present study evaluated differences in cadmium, calcium, copper, manganese, magnesium, nickel, lead, and zinc levels in red crabs between years with and without El Niño conditions. We also evaluated whether the composition of these elements varies between sites along the coast of the Baja California Peninsula and the potential risk to human health if red crab is consumed as food. Cruises showed significant differences in the content of trace and macro elements, mainly associated with environmental variables that influence the vertical distribution of the red crab from 51 m to 500 m. Red crab provides less than 2% of the nutrient requirements of calcium, copper, manganese, magnesium, and zinc in the human diet; in addition, the contribution of cadmium, lead, and nickel recorded in this study poses no risks to human health.

**Abstract:**

The red crab, *Pleuroncodes planipes*, is a decapod crustacean abundant off the Pacific coast of the Baja California Peninsula. This species is caught and used in preparing animal feed, such as flour, particularly for aquaculture. Levels of calcium (Ca), cadmium (Cd), copper (Cu), iron (Fe), lead (Pb), magnesium (Mg), manganese (Mn), nickel (Ni), phosphorus (P), and zinc (Zn) were measured in red crabs collected from three geographic zones during three cruises in different seasons. Significant differences were found in the levels of Ca, Cd, Cu, Fe, Mg, Ni, P, and Zn between the two El Niño years (cruises C1 and C3, based on a threshold of ±0.5 °C for the Oceanic Niño Index). The highest concentrations of most elements were observed in the south of the Baja California Peninsula, a highly productive area influenced by upwelling events. Our findings suggest that while environmental temperature plays a central role in the benthic or pelagic distribution of red crabs, their content and variability of trace and macro elements appear to be associated with the presence of oceanic conditions, such as upwelling and potential changes in the composition of their diet associated with the depth in which these crustaceans are collected.

## 1. Introduction

Zinc (Zn), copper (Cu), manganese (Mn), iron (Fe), calcium (Ca), magnesium (Mg), and phosphorus (P) are essential elements required to perform different metabolic processes. Consequently, their levels and the biochemical mechanisms that regulate these levels in the organism vary across species [1]. When in excess, chemical elements can have adverse health effects, and these trace elements have been associated with severe effects in animals, including humans [1,2,3]. The chemical composition and bioavailability of trace elements in the aquatic environment and the organisms that thrive in it are influenced by the local geochemistry (volcanism, mineral deposits) and anthropogenic activities (agriculture, mining) that can introduce high amounts of metallic and non-metallic elements [1]. One of the largest phosphorite deposits worldwide is found on the coast of the Baja California Peninsula. Common impurities in phosphate-rich rocks are cadmium (Cd), lead (Pb), Cu, and Zn [4,5]. In addition, in the Pacific Ocean off the Baja California Peninsula, there are sites where upwelling events contribute to enriching elements such as Cd and P in the water column [6].

The red crab *Pleuroncodes planipes* Stimpson, 1860 (Decapoda, Anomura, Galatheoidea) is abundant in the Pacific coast upwelling system of the Baja California Peninsula [7]. During the first year of life, *P. planipes* is part of the micronekton [8]. In the larval and juvenile stages, it is found in pelagic and benthic environments, while the adult stage (upon 32 mm length) becomes strictly benthic, found at depths between 200 m and 500 m [8,9,10]. Depending on the conditions in the ocean, the feeding habits of this crab vary due to its mass bathymetric migration.

The red crab is a key food item for many predators, including cetaceans, pinnipeds, birds, turtles, and fishes [9,11], being an important component in the trophic web in the Pacific Ocean. The estimated annual abundance of the red crab along the Pacific coast of the Baja California Peninsula was 460,217 tons in 1995 [12], increasing to 611,525 tons by 2014 [13]. In addition to its ecological importance, this species is a major economic resource. Once red crabs are collected, they are processed (whole, without further preparation) to obtain flour used as a feed ingredient for cultured shrimp. This flour has been evaluated with encouraging results [7,14,15]. Dried whole red crab contains about 43% protein, 8% lipids, and 7.1% astaxanthin [16]. Given the need to find protein sources for humans, red crab flour could be included as a food ingredient for human nutrition [17].

The red crab feeds mainly on organic matter (approximately between 60% and 70%) and the composition of its diet can vary over time and between sites [8,12]. The oceanic conditions vary between years, inducing major faunal shifts [18], mainly changes in zooplankton abundance [19], that affect the marine trophic ecology and lead some species to use alternative food sources. Aurioles-Gamboa et al. [8] found that in winter, red crabs are distributed across the entire continental shelf. However, in summer, they are found only on the outer shelf (100–200 m depth), and their stomach contents are markedly lower than those recorded during winter at the same depths; these authors concluded that this pattern is correlated with the lower intensity of the coastal upwelling system and the weakening of the California Current. Also, the red crab nutritional status (fatty acid, lipid and astaxanthin contents) varies across feeding sites [7]. Therefore, if the nutrient content of red crabs varies over time and seasonally, the content of chemical elements may also vary. Bioaccumulation of trace metals in marine organisms can eventually lead to adverse health effects and can be a potential health risk to humans if contaminated seafood is consumed in the human diet [20].

Red crabs are used mainly for animal feed, as they are a protein source, and they could also be used for human consumption. Seafood is a good source of animal protein for humans because it usually contains all of the essential amino acids and also has a low fat content [21]. An increasing trend to include seafood in the human diet has been reported; for example, the average annual European Union consumption per person is 21.97 kg [17]. Trace-metal concentrations in commercial marine organisms should be evaluated for seafood safety and public health reasons, as well as for sustainable management of the coastal environment [22,23]. Therefore, the objective of this study was to assess temporal and spatial variations in the content of trace and macro elements in red crabs.

## 2. Materials and Methods

### 2.1. Study Area

**Figure 1:**
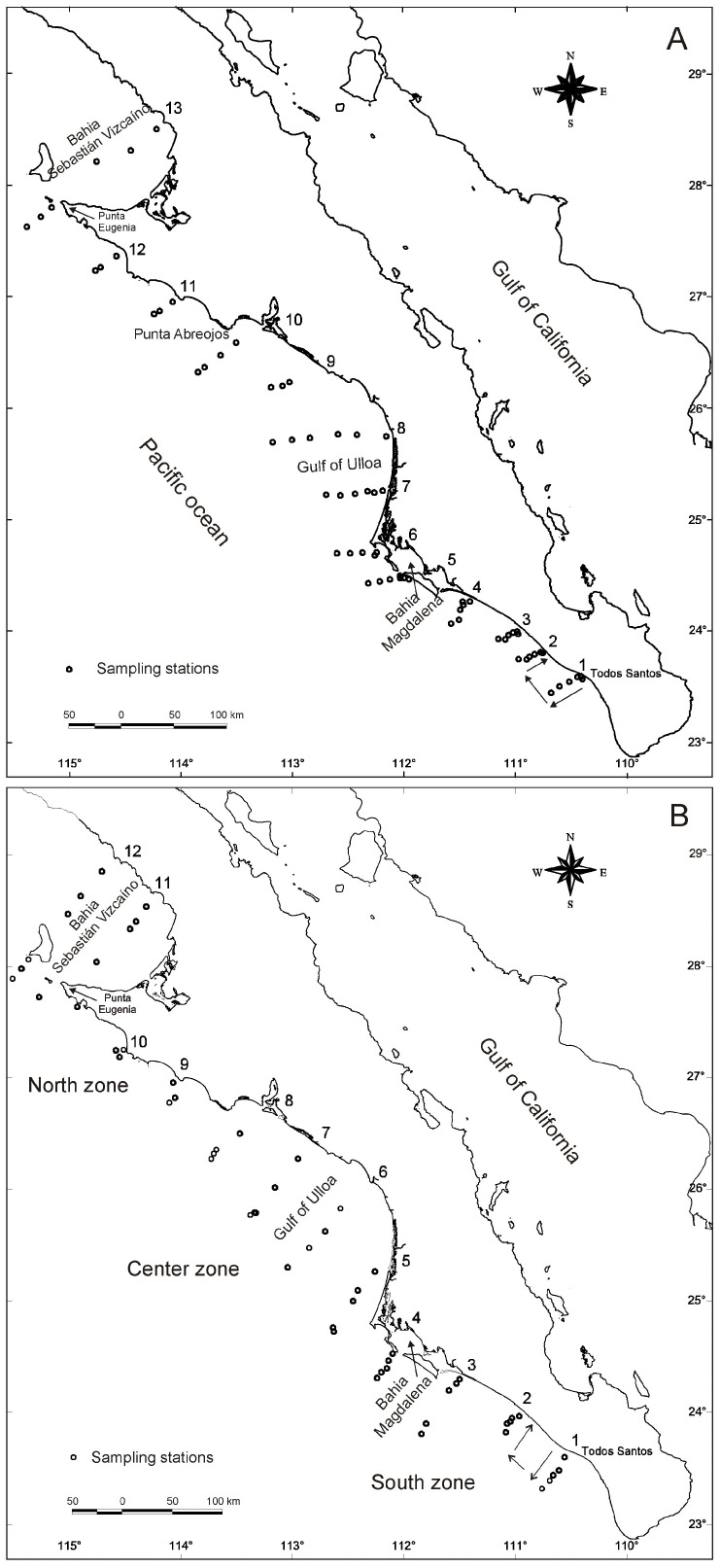
Geographic location of the sampling stations of the research cruises along the west coast of the Baja California Peninsula, Mexico. (**A**) Cruise 1, October–November 2004; (**B**) Cruise 2, March 2005; (**B**) Cruise 3, November 2006. The numbers over the peninsula indicate the transect number.

The study area (latitude 23°19′ N–longitude 110°45′ W and latitude 28°51′ N–longitude 114°42′ W) is located in the Pacific Ocean, off the west coast of the Baja California peninsula. In this area (Figure 1A,B), three research cruises were conducted onboard the research vessel “BIP XII”. The first cruise (C1) took place between 21 October and 10 November 2004 (Figure 1A). Autumn of 2004 was considered to be under the influence of an El Niño event, lasting from June 2004 to February 2005 based on a threshold of ±0.5 °C in the Oceanic Niño Index (ONI; http://www.cpc.ncep.noaa.gov/products/analysis_monitoring/ensostuff/ensoyears.shtml; accessed on 8 march 2015). The second cruise (C2) occurred from 15 March to 29 March 2005 (Figure 1B). Spring of 2005 was declared a neutral year (see the website above). The third cruise (C3) was conducted between 21 November and 4 December 2006 (Figure 1B). Autumn of 2006 was considered to be under the influence of an El Niño event. The oceanographic dynamics of the west coast of the Baja California peninsula are influenced primarily by the California Current, the California Subcurrent, and the North Equatorial Current. The prevailing winds are strong in coastal zones, triggering cold upwelling events that foster plankton blooms and abundant sea life, including species that are important for fisheries [24,25,26,27].

### 2.2. Sampling

Sampling was performed following a stratified random design along evenly spaced transects (of variable length, depending on the topography) running perpendicular to the coast (Figure 1). At each station, bottom trawls were performed as described by [13,28]. The region in which red crabs were collected was split into three zones: south, center, and north (Figure 1B). The transects sampled at each site were used as replicates of a zone for statistical analyses (south: Todos Santos–Bahía Magdalena; center: Gulf of Ulloa; north: Punta Abreojos–Bahía Sebastián Vizcaíno). The specimens of *P. planipes* collected were stored at −20 °C until analyses.

### 2.3. Analysis of Trace and Macro Elements

When red crab is used as feed for aquaculture, specimens are processed whole, i.e., the viscera, muscle, and shell are not separated to produce the flour used to feed aquatic organisms. For this reason, a total number of 279 whole adult specimens were selected and analyzed, with body size ranging between 32 mm and 40 mm; samples were dried for 72 h at 70 °C. For the analysis of trace and macro elements, specimens were subjected to acid digestion in nitric acid (HNO_3,_ concentrated) and hydrogen peroxide (H_2_O_2_, 30%) in Teflon vials using a microwave oven (Mars 5x, CEM, Matthews, NC, USA). The concentrations of Ca, Cd, Cu, Fe, Mg, Mn, Ni, Pb, and Zn were quantified using an atomic absorption spectrophotometer (GBS Scientific AVANTA, Dandenong, Australia) with air–acetylene flame [22,29]. Phosphorus (P) content was measured with the molybdovanadate method [30], which was validated using blanks tested in parallel [31]. High-purity reagents were used throughout the testing process. Standardized reference material (TORT-2, DORM-2 and DORM-4 from the National Research Council of Canada; Ottawa, Canada) samples spiked with known concentrations of trace elements and macro elements, and blanks were also analyzed as a quality control [32]. The analyses of metal content yielded recovery values ranging from 93% to 116% for the entire process. The highest recovery value corresponded to magnesium, which probably included the contribution of air pollution. The runs by element analyzed were for triplicate. The limits of detection and of quantification (µg g^−1^), respectively, were as follows: Ca: 0.08 and 0.10; Cd: 0.01 and 0.02; Cu: 0.01 and 0.02; Fe: 0.65 and 1.35; Mg: 0.05 and 0.08; Mn: 0.04 and 0.07; Ni: 0.03 and 0.05; Pb: 0.02, and 0.07; Zn: 0.02 and 0.06. All the concentrations are expressed in dry weight.

### 2.4. Statistical Analyses

The datasets were tested for normality and homoscedasticity using the Shapiro–Wilk and Levene tests. To explore significant differences in the content of trace elements (Cd, Cu, Fe, Mn, Ni, Zn) and macro elements (Ca, Mg, and P), concentrations were grouped by cruise (C1, C2, and C3) and zone (south, center, north). To this end, trace element concentrations were log-transformed. For elements recorded at concentrations below the limit of detection, the value corresponding to one-half of the respective limit of detection was used for statistical analyses [33]. Since the number of samples differ between cruises and zones, the analyses were carried out by testing the Type-1 hypothesis (Type-1 decomposition), which is particularly useful for complex unbalanced designs [34]. Statistical significance was defined at *p* < 0.05. Data for all variables met the normality and homoscedasticity assumptions, except for P (usually, slight deviations from the normality and homoscedasticity assumptions do not bias the *F*-test). Lindman [35] showed that the *F* statistic is quite robust against deviations of the homoscedasticity assumption. However, P failed to comply with the assumption of no correlation between means and standard deviations. A two-way analysis of variance (ANOVA) was performed, using cruise and zone as fixed main factors and including one two-way interaction term, followed by a comparison of cruises C1 and C3 (El Niño) combined vs. C2 (non-El Niño). Bonferroni’s post hoc test was used to determine significant differences between group means [36]. Discriminant analysis followed by a Factor analysis was carried out to identify how the different trace and macro elements grouped together in each cruise. The variable “zone” as ordinal variable was transformed to numerical value (South = 1; Central = 2; North = 3) and included in the factorial analysis according to Robitzsch [37]. All statistical analyses were run using the software STATISTICA version 13.5.0.17 [34].

### 2.5. Health Risk

The results obtained in this study were compared with guidelines and results of previous studies to assess the potential health risk of human consumption of red crabs. With this purpose, element concentrations were converted from dry weight (dw) to fresh weight (fw) as follows:

Element fw = Element dw × (100 − % moisture)/100(1)

using the percentage of moisture in red crabs (range 73.8–79.4%). The estimated daily intake (EDI, mg trace element kg^−1^ BW day^−1^) was calculated as follows [38]:

EDI = (Cm × CR)/BW(2)

where Cm = mean concentration of the chemical element in red crabs, expressed as a fresh weight (µg g^−1^); CR = mean daily per-capita consumption rate of crabs (41 g day^−1^ per person); BW = mean body weight of an adult person (70 kg) [17]. From a nutritional standpoint, we used the recommended daily intake (RDI) [39] for Fe (8 mg day^−1^), Zn (11 mg day^−1^), Cu (0.9 mg day^−1^), Mn (2.3 mg day^−1^), Ca (1000–1200 mg day^−1^), Mg (400–420 mg day^−1^), P (700 mg day^−1^), and a tolerable intake of Ni (1.0 mg day^−1^) to value the contribution of the estimated RDI for these elements. No established RDI has been set for cadmium and lead because these metals are considered toxic to humans. To measure the possible toxicological risk of the intake of the elements mentioned, from the consumption of red crabs, each EDI was compared with its respective reference oral dose (RfD): Cd: 1.0 μg kg^−1^ body weight day^−1^; Cu: 40.0 μg kg^−1^ bodyweight day^−1^; Fe: 700 μg kg^−1^ bodyweight day^−1^; Mn: 0.140 μg kg^−1^ bodyweight day^−1^; Ni: 20.0 μg kg^−1^ bodyweight day^−1^; and zinc: 300 μg kg^−1^ bodyweight day^−1^ [40]. The tolerable upper intakes (DRIs) for Ca were been set as 2500 g day^−1^; for Mg: 350 g day^−1^; and for P: 4.0 g day^−1^ [39].

## 3. Results

No detectable levels of Pb (<0.07 µg g^−1^) were found in red crabs from any location or cruise. Thus, this element was not included in the statistical analyses. The results of the multivariate test of trace (Cd, Cu, Fe, Mn, Ni, Zn) and macro element (Ca, Mg, P) concentrations showed significant differences in the different groups (cruise and zone), including their interaction (*p* < 0.05) (Table 1).

Post hoc comparisons of means with the Bonferroni test (Table 2) showed significant differences in Cd, Zn, Cu, Fe, and Mg content between C1 vs. C2. In addition, significant differences were observed in Zn, Cu, Ni, Fe, Ca, and P between C2 vs. C3, as well as in Cd, Zn, Cu, Ni, Fe, Ca, Mg, and P levels between the two El Niño years (C1 and C3). Although both C1 and C3 showed El Niño conditions (positive anomalies of up to 0.9 °C and 1.1 °C, in October–November 2004 and November 2006, respectively; NOAA data), the highest concentrations of Cd and Zn, significantly different (*p* < 0.05) compared to C3, were recorded in red crabs collected during C1, and Ni and Mg content was significantly higher in C3 (Table 2). On the other hand, the highest significant (*p* < 0.05) concentrations of Cu, Fe, Ca, Mg, and P were recorded in C2 (normal environmental conditions with positive temperature anomalies of 0.3 °C in March 2005; NOAA data), although Mg content in C2 was not significantly different from the concentration recorded in C3, and neither were Ca and P concentrations in C2 versus C1 (Table 2).

Spatially (south, center and north), the lowest Cd and Fe content was found in the north (Punta Abreojos—Bahía Sebastián Vizcaino). The lowest concentrations of Zn, Ca, and Mg were found in the center, and the highest concentrations of Cu, Mn, Fe in the south (Todos Santos—Bahía Magdalena). In addition, the highest Cd levels (up to 23.10 µg g^−1^) were found in the south. Ni and P content did not show significant differences between the three zones. The central zone (Golfo de Ulloa) generally showed the lowest concentrations of all the trace and macro elements analyzed in red crabs during the three cruises (C1, C2, and C3), especially towards the south (Table 3).

### 3.1. Discriminant Analysis

All trace and macro element concentrations included in the discriminant function analysis were highly significant in discriminating between the cruises (Wilks’ Lambda = 0.149; *F* = 47.30; *p* < 0.000; Table 4). The first root distinguished C1 and C3 and explained 91% of the cumulative variance in elemental concentrations; the variables with the greatest contribution were Mn, Ni, Fe, Ca, Mg, and P content (Table 5). In addition, the second root differentiated cruises C1 and C2 and explained 9% of the cumulative variance in elemental concentrations; the contributing variables were Cd, Zn, and Cu levels, with a relatively high correlation between the concentrations of Cd and Cu (0.44) (Table 5). The highest levels of these elements were recorded in crabs from the south zone. However, only the highest concentrations of Cd and Cu coincided with the fact that they were found in organisms from C1 and C2, respectively.

**Figure 2:**
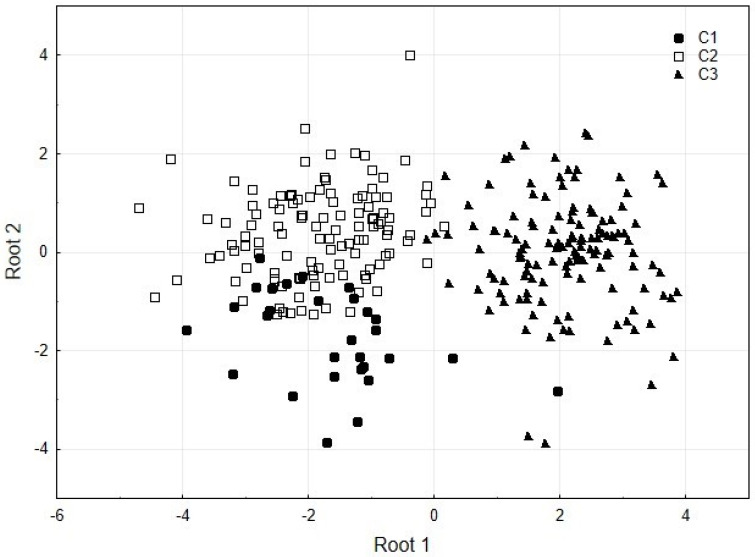
Scatterplot of canonical scores for two discriminant functions (roots) between cruises (C1–C3).

The canonical scores, the discriminant function mainly differentiated between C1 and C2; the distribution of the values for C1 are below the central line (0) with a mean of −1.73, while C2 values show a mean of 0.40 (Figure 2). These results discriminate clearly and significantly between C3 and C1-C2, and between C1 and C2 with the first and second discriminant functions, respectively.

### 3.2. Factor Analysis

**Figure 3:**
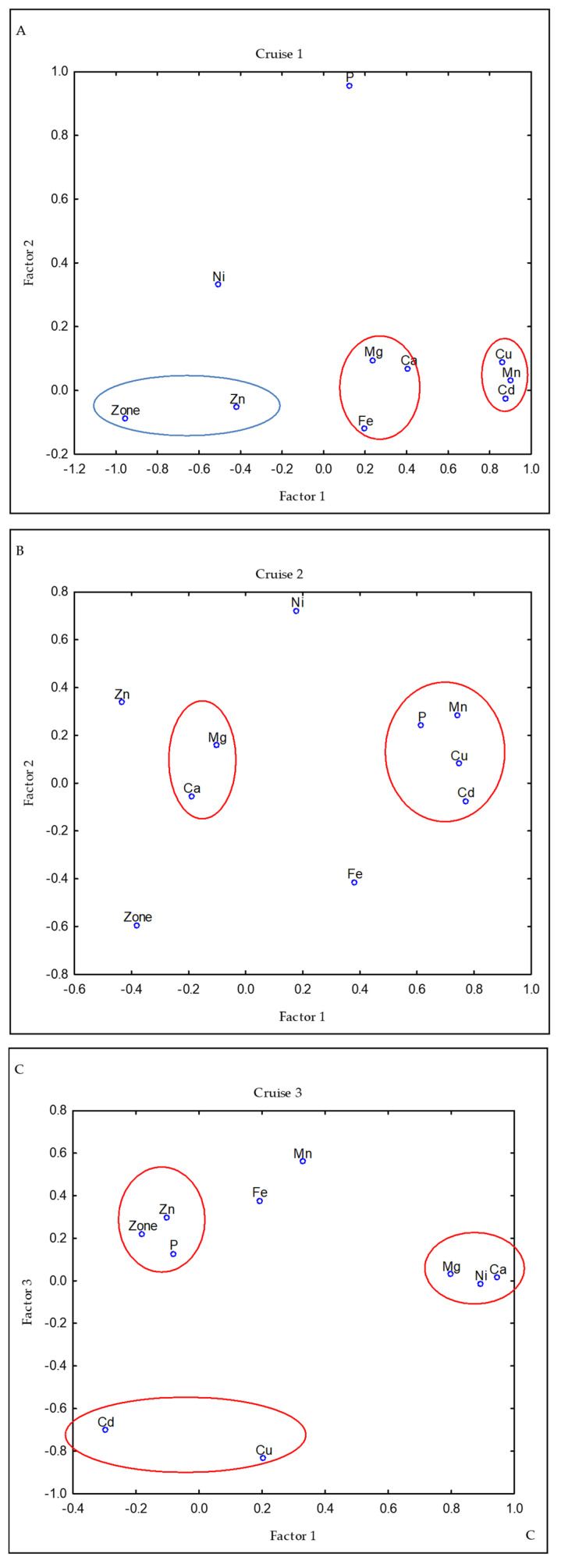
Loadings of trace and macro elements in red crab by a cruise on varimax rotate using factor analysis. The variables with significant loadings to each factor were grouped. In C1 (**A**), the elements in the blue circle have an inverse relationship with the elements in the red circle. No inverse relationship were recorded in C2 (**B**) or C3 (**C**).

In the red crab, the loadings of the trace and macro elements that were assessed varied across cruises (Table S1 in Supplementary Material). In the first cruise (C1), the first three factors accounted for 84.4% of the variance, while in cruises 2 and 3 (C2 and C3), the first three factors explained 65.3% and 64.5% of the variance, respectively (Table S1). However, the groups and loadings of some elements in the factors that were derived from the factor analysis were constant: Cd, Cu, and Mn were the elements with the greatest loadings to Factor 1 in C1 and C2. In C3, these three elements were associated with Factor 2, where Ca and Mg are the elements with the greatest loadings, followed by Mn. In C1 (Figure 3A), an inverse relationship was recorded between the group formed by Mg, Ca, and Fe (red circle) vs. the zone and Zn (blue circle); that means the highest concentrations of Mg, Ca, and Fe but the lower of Zn were in the south during C1. The other elements did not show a consistent association with the other elements, nor were their greatest loadings found in the same factor during the cruises. For its part, Fe contributed to Factor 2 along with Ca, Mg, and Zn in C1, and to Factor 3 along with P and Zn in C3, but showed no significant loading related to any factor in C2. In C3, Fe and slightly P showed an inverse contribution with the zone; zone 1 showed the highest Fe and P concentrations, while the lowest Fe and P concentrations were grouped together in zone 3. In addition, in C3, Zn slightly showed a direct contribution to the zone.

### 3.3. Health Risk Assessment

In relation to the % RDI of trace elements associated with the consumption of 41 g of red crab, the contribution of these elements to the human diet is low (<1%), Cu being the element with the highest contribution (~0.7%), followed by Fe (~0.2%), Zn (~0.09%), Mn (~0.03%), and Ni (~0.03%) (Table 6). In addition, the contributions of these five elements plus Cd and Pb (the concentrations of the latter below detection limits) from the consumption of red crab are several orders of magnitude below the RfD established for each specific element (Table 6).

As for the macro elements, Ca concentrations in red crab contribute approximately 1% to the recommended RDI for a 70 kg person, followed by magnesium with 0.3% and phosphorus with 0.2% (Table 6).

## 4. Discussion

Except for Cd, the concentrations of the elements measured in red crabs collected in all cruises, particularly Pb (below 0.07 µg g^−1^), were within the normal range observed in organisms living in non-polluted areas and, therefore, the levels of these trace elements were not of dietary concern [41,42,43,44]. Although the Cd concentrations recorded in this study may seem high compared with the content recorded in other marine organisms, they are within the range considered normal for crabs [45], in which values as high as 13 µg g^−1^ [44,46] and even up to 50 µg g^−1^ wet weight [47] have been reported. Red crabs are found mainly in areas influenced by upwelling events because these provide a rich source of nutrients and optimum water conditions (below 16 °C) for this species [8,10]. During upwelling events, particulate material enriched with Ca, Cu, and especially Cd [6,48] is mobilized from the bottom to the surface of the water column [6,9]. Particulate matter, which accounts for about 60% to 70% of the diet of red crabs, contains diatoms [49]. This is relevant because up to 17 genera of diatoms present in plankton have been reported in the stomach contents of red crabs [8]. The high Cd content found in red crabs can be associated with the consumption of diatoms, which are a major source of Cd [50,51,52]. Although Cd is bioaccumulated by *P. planipes*, this species has the capacity to make it non-bioavailable through detoxification mechanisms that include Cd binding to high-affinity low-molecular-weight proteins known as metallothioneins [47]. Along with Cd, the highest Cu and Mn concentrations in red crab were found in the south, an area characterized by submarine phosphorite deposits [53]. In phosphate rocks, Cd, Cu, Ni, and Mn are common impurities [4,5,54]. Cu is a particularly important element for crustaceans for being a component of hemocyanin, the protein responsible for transporting oxygen in the metabolism of these organisms. The factor analysis showed that Cd and Cu grouped together in the different factors. Cu metabolism also involves metallothioneins, as their synthesis can be stimulated by Cu homeostasis [55]. The importance of Cu in crustaceans likely explains the fact that it is the trace element supplied in highest levels to the human diet. Cd is another element present at high concentrations in red crabs. Cadmium has similar metabolism pathways to copper in these organisms, which poses a potential risk for human health given its toxicity. However, the amount of trace elements contained in 41 g of red crab is lower than 1%, which poses a low risk from its consumption by humans. After copper, Fe is the second most important trace element for its content in red crabs. In the present study, the results of the factor analysis show that in C3, Fe and Zn content in these organisms may be associated with the area where they were collected. Additionally, the highest Fe concentrations were also found in red crabs collected in the south zone. Fe is an indicator of both terrigenous materials and the resuspension of sediments from the continental shelf [56], which can also be associated with upwelling events. De Anda-Montañez et al. [13,29], in a spatio–temporal analysis of red crabs collected in the same cruises, reported that during the spring of 2005 (C2) under environmental conditions of a non-El Niño year, red crabs were highly abundant and widely distributed from 51 m to 300 m depth. The red crabs collected during C2 (in spring) recorded the highest Cu, Fe, Ca, Mg, and P levels relative to the other cruises. This finding is likely due to the fact that the most intense upwelling period occurs in spring and early summer (March–June), although upwelling events occur less frequently throughout the year in the area studied in this work [57]. C1 and C3 were carried out in autumn under El Niño conditions. In these cruises, red crabs were collected at 200 m depth in C1, in contrast with C3, when the abundance and distribution of *P. planipes* increased at 400 m to 500 m depth [13,29]. El Niño events are associated with a shift in salinity and thermal stratification along the water column [53,54]. In general, a linear and significant relationship was observed, that is, higher temperatures were associated with lower red crab catches [13,29]. *P. planipes* alternates between pelagic and benthic environments according to seawater temperature [9], migrating to deeper layers during El Niño years [13,29]. Therefore, environmental variability plays a central role in the distribution of *P. planipes* and the variability of its content of trace and macro elements. Although El Niño conditions prevailed during C1 and C3, the concentrations of Cd, Zn, Ca, and P were lower in crabs collected during C3, when these organisms were found at deeper layers than in C1 [29]. Zooplankton and inorganic matter are the main components in the diet of *P. planipes* collected at depths of 150 m to 200 m [8]. In the study area, zooplankton biomass and chemical composition are influenced mainly by the California Current that converges in upwelling areas [57]. Elements with potentially enriched concentrations in these areas, such as Fe and Mg, interfere with the absorption of P due to the formation of insoluble phosphate salts [55]. In addition, other food components, such as lipids, can affect Ca and P uptake [56]. Robinson et al. [57,58] reported that 90% of the total abundance of red crabs in their study was found in areas where chlorophyll-*a* concentrations increased as a result of upwelling events, which are a major source of trace metals, and where submarine phosphorite deposits are present [53].

## 5. Conclusions

Most of the significant differences observed in the levels of trace and macro elements in red crabs were associated with environmental changes that probably influence the diet of this crustacean species, such as zooplankton abundance. The organisms collected during C3 (El Niño year), when red crabs were found at deeper levels, recorded the lowest Ca, P, and Zn content. The highest Cd, Cu, Mn, Ni, Fe, and P levels were recorded in the south, probably due to the presence of submarine phosphorite deposits and upwelling events, which boost primary productivity. Although the content of trace and macro elements changes in organisms, some associations between elements were observed in the three cruises, such as those between Ca and Mg, and between Cd and Cu. In contrast, Fe, Ni, Mn, P, and Zn showed no consistent associations with other elements in the three cruises.

Previous studies show that flour from red crabs is a protein source. Our findings suggest that although its consumption poses no risks to human health, it is not an important source of minerals of nutritional value since 41 g of red crab consumed per day represent less than 1% of the recommended daily dose of trace elements of nutritional value. For the consumption of red crab to pose a human health risk associated with Cd toxicity, a much higher amount of this crustacean would have to be consumed. As for the macro elements, Ca concentrations in red crab contribute approximately 1% to the recommended RDI for a 70 kg person, followed by magnesium with 0.3% and phosphorus with 0.2%. Continued monitoring of the contents of trace and macro elements in red crabs is recommended to guarantee the safe consumption of this crustacean by humans.

## Figures and Tables

**Table 1:** Multivariate significance tests of the concentrations of trace and macro elements in red crabs, *Pleuroncodes planipes*, using cruise and zone as fixed main factors, including one 2-way interaction term (combined effect: Cruise × Zone).

Effect	Test	Value	*F*	Effect df	Error df	*p*
Intercept	Wilks	0.000085	341071.4	9	262.0	0.00
Cruise	Wilks	0.114642	56.9	18	524.0	0.00
Zone	Wilks	0.470140	13.3	18	524.0	0.00
Cruise × Zone	Wilks	0.154107	17.7	36	983.6	0.00

**Table 2:** Concentrations of trace and macro elements in red crabs, *Pleuroncodes planipes*, collected during three cruises (C1, C2, C3) off the west coast of the Baja California Peninsula, Mexico. Data are shown as mean plus/minus standard error (SE), with the concentration interval in parenthesis. Concentrations are expressed in dry weight and N is sample size per cruise. Rows with different superscript letters denote statistically significant differences (*p* < 0.05).

Cruise	C1 (N = 29) Mean ± SE	C2 (N = 119) Mean ± SE	C3 (N = 131) Mean ± SE
Cadmium (µg g^−1^)	12.07 ± 0.76 ^a^	9.27 ± 0.24 ^b^	8.97 ± 0.28 ^b^
	(8.01–23.1)	(0.24–18.1)	(0.12–20.9)
Zinc (µg g^−1^)	71.34 ± 3.04 ^a^	63.00 ± 0.76 ^b^	56.88 ± 1.03 ^c^
	(52.4–111)	(44.7–84.7)	(39.9–131)
Copper (µg g^−1^)	40.46 ± 4.44 ^a^	49.35 ± 1.75 ^b^	41.21 ± 1.03 ^c^
	(15.1–92.3)	(27.5–88.5)	(18.0–82.1)
Manganese (µg g^−1^)	4.47 ± 0.58 ^a^	6.64 ± 0.57 ^a^	4.92 ± 0.33 ^a^
	(<0.04–10.2)	(<0.04–27.7)	(<0.04–17.4)
Nickel (µg g^−1^)	1.23 ± 0.26 ^a^	1.56 ± 0.16 ^a^	3.19 ± 0.23 ^b^
	(<0.03–4.43)	(<0.03–6.52)	(<0.03–13.79)
Lead (µg g^−1^)	<0.07 ^a^	<0.07 ^a^	<0.07 ^a^
Iron (µg g^−1^)	127 ± 11 ^a^	162 ± 5 ^b^	111 ± 8 ^c^
	(54.1–247)	(45.3–389)	(21.8–532)
Calcium (mg g^−1^)	8.41 ± 0.22 ^a^	9.05 ± 0.007 ^a^	7.27 ± 0.12 ^b^
	(5.84–10.7)	(4.34–12.0)	(4.59–12.7)
Magnesium (mg g^−1^)	0.97 ± 0.03 ^a^	1.09 ± 0.02 ^b^	1.09 ± 0.01 ^b^
	(0.68–1.25)	(0.53–1.46)	(0.84–1.46)
Phosphorus (mg g^−1^)	0.97 ± 0.04 ^a^	1.13 ± 0.02 ^a^	0.67 ± 0.02 ^b^
	(0.06–1.77)	(0.75–2.59)	(0.4–1.10)

**Table 3:** Concentrations of trace and macro elements in red crabs, *Pleuroncodes planipes*, collected and grouped by zone (south, center, and north) during three cruises (C1, C2, C3) off the west coast of the Baja California Peninsula, Mexico. Data are shown as mean plus/minus standard error (SE), with the concentration interval in parenthesis. Concentration is expressed in dry weight. N is sample size per zone. Rows with different superscript letters denote statistically significant differences (*p* < 0.05).

Zone	South (N = 142) Mean ± SE	Center (N = 81) Mean ± SE	North (N = 56) Mean ± SE
Cadmium (µg g^−1^)	10.10 ± 0.32 ^a^	9.31 ± 0.28 ^a^	7.84 ± 0.19 ^b^
	(0.12–23.1)	(4.95–20.9)	(4.36–11.0)
Zinc (µg g^−1^)	61.58 ± 0.99 ^a^	57.32 ± 0.77 ^b^	64.82 ± 2.12 ^a^
	(39.9–131)	(40.1–74.8)	(43.6–111)
Copper (µg g^−1^)	48.78 ± 1.26 ^a^	39.35 ± 1.33 ^b^	41.61 ± 1.75 ^b^
	(18.0–92.3)	(20.0–82.2)	(15.1–71.0)
Manganese (µg g^−1^)	7.59 ± 0.46 ^a^	4.20 ± 0.45 ^b^	2.60 ± 0.19 ^b^
	(0.06–27.7)	(<0.04–15.3)	(<0.04–5.63)
Nickel (µg g^−1^)	2.70 ± 0.23 ^a^	1.91 ± 0.21 ^a^	1.81 ± 0.23 ^a^
	(<0.03–13.8)	(<0.03–7.3)	(<0.03–6.7)
Lead (µg g^−1^)	<0.07 ^a^	<0.07 ^a^	<0.07 ^a^
Iron (µg g^−1^)	155 ± 7 ^a^	100 ± 6 ^b^	134 ± 10 ^c^
	(38.4–532)	(21.8–247)	(32.4–389)
Calcium (mg g^−1^)	8.22 ± 0.13 ^a^	7.32 ± 0.13 ^b^	9.17 ± 0.24 ^a^
	(4.34–12.7)	(4.59–10.5)	(5.45–12.0)
Magnesium (mg g^−1^)	1.09 ± 0.01 ^a^	1.04 ± 0.01 ^b^	1.12 ± 0.01 ^a^
	(0.53–1.46)	(0.68–1.30)	(0.61–1.40)
Phosphorus (mg g^−1^)	0.95 ± 0.03 ^a^	0.82 ± 0.02 ^a^	0.91 ± 0.02 ^a^
	(0.5–2.58)	(0.06–1.8)	(0.5–1.6)

**Table 4:** Chi-square tests with successive roots removed.

Roots Removed	Eigenvalue	Canonical R	Wilks’ Lambda	*X* ^2^	d.f.	*p*-Value
0	3.847	0.891	0.149	517.37	18	0.000
1	0.382	0.526	0.723	88.06	8	0.000

**Table 5:** Raw coefficients for canonical variables. An asterisk denotes the variables with the greatest contribution to each root.

Variable	Root 1	Root 2
Cadmium	0.005	−0.878 *
Zinc	−0.271	−0.484 *
Copper	−0.187	0.527 *
Manganese	0.318 *	0.289
Nickel	0.469 *	−0.204
Iron	−0.422 *	0.181
Calcium	−1.401 *	−0.289
Magnesium	1.081 *	0.570
Phosphorus	−0.744 *	0.215
Eigenvalue	3.847	0.382
Variance explained	0.910	1.000

**Table 6:** Contribution of trace and macro elements from the consumption of *Pleuroncodes planipes*, (mg day^−1^) relative to the recommended daily intake (RDI) (mg day^−1^, [39]) and reference dose RfD (μg kg^−1^ bodyweight day^−1^, [40]).

Element	EDI	Calculated RDI	Theoretical RDI	% RDI	RfD	% RfD
Cadmium	1.33 × 10^−03^		na		0.07	1.90 × 10^+00^
Copper	6.31 × 10^−03^	2.05 × 10^−04^	0.9	0.70	2.8	2.25 × 10^−01^
Iron	1.90 × 10^−02^	7.32 × 10^−04^	8	0.24	49	3.88 × 10^−02^
Manganese	7.86 × 10^−04^	5.42 × 10^−05^	2.3	0.03	9.8	8.02 × 10^−03^
Nickel	2.96 × 10^−04^	2.34 × 10^−05^	1.0	0.03	1.4	2.11 × 10^−02^
Zinc	9.59 × 10^−03^	1.70 × 10^−04^	11	0.09	21	4.57 × 10^−02^
Calcium	1.23 × 10^+01^	3.10 × 10^−01^	1200	1.03	na	
Magnesium	1.57 × 10^+00^	2.98 × 10^−02^	420	0.37	na	
Phosphorus	1.40 × 10^+00^	4.29 × 10^−02^	700	0.20	na	

na: not available.

## Data Availability

The data presented in this study are available on request addressed to Dr. Juan Antonio de Anda-Montañez (jdeanda@cibnor.mx (J.A.D-M)).

## Supplementary Materials

The following supporting information can be downloaded at: https://www.mdpi.com/article/10.3390/ani13050822/s1, Table S1: Loadings of trace and macro elements in red crab by cruise on varimax rotate using factor analysis. The variables with the greatest loadings to each factor are market with an asterisk.
