# Supplementary material for: Spatial and Temporal Variability of Trace and Macro Elements in the Red Crab Pleuroncodes planipes in the Pacific Coast of the Baja California Peninsula, Mexico
Source: Animals (Basel). 2023 Feb 24;13(5):822. doi: 10.3390/ani13050822 (PMC10000037; doi:10.3390/ani13050822)
Supplement: Supplementary file 1 [file animals-13-00822-s001.zip › animals-2201859-supplementary.pdf]

animals

MDPI

Supplementary Material

Table S1. Loadings of trace and macro elements in red crab by cruise on varimax rotate using factor analysis. The variables with the greatest loadings to each factor are market with an asterisk.

Variable	Cruise 1			Cruise 2			Cruise 3		
	Factor 1	Factor 2	Factor 3	Factor 1	Factor 2	Factor 3	Factor 1	Factor 2	Factor 3
Zone	- 0.95*	0.16	- 0.09	- 0.38	0.40	- 0.60	- 0.18	0.22	- 0.72*
Calcium	0.41	0.81*	0.07	- 0.19	0.90*	- 0.06	0.95*	0.01	0.00
Cadmium	0.88*	0.17	- 0.03	0.77*	- 0.24	- 0.08	- 0.30	- 0.70*	0.07
Copper	0.87*	0.33	0.09	0.75*	0.24	0.08	0.21	- 0.84*	- 0.12
Iron	0.20	- 0.86*	- 0.12	0.38	0.43	- 0.42	0.19	0.37	0.72*
Magnesium	0.24	0.88*	0.09	- 0.10	0.90*	0.15	0.80*	0.03	0.09
Manganese	0.90*	- 0.23	0.03	0.74*	- 0.38	0.28	0.33	0.56	0.24
Nickel	- 0.50	- 0.56	0.33	0.18	0.39	0.72*	0.90*	- 0.02	0.10
Phosphorus	0.13	0.15	0.95	0.62*	- 0.38	0.24	- 0.08	0.13	- 0.61*
Zinc	- 0.41	0.83*	- 0.05	- 0.43	0.19	0.34	- 0.10	0.29	0.69*
Exp.Var.	3.96	3.41	1.07	2.64	2.55	1.34	2.66	1.80	1.99
Exp. Var Var. (%)	39.6	73.7	84.4	26.4	51.9	65.3	26.6	44.6	64.5

Exp. Var.= Explained variance; Var.= Variance (in percentage).
